# Patient perceptions of artificial intelligence integration in dermatology: a cross-sectional study of trust, comfort and equity across multiple care modalities

**DOI:** 10.1093/skinhd/vzaf086

**Published:** 2025-12-22

**Authors:** Charlotte McRae, Michael Anderson, Laci Turner, Alexandra Savage, Saleem Mohammad, Rachael Cowan, Lauren V Graham

**Affiliations:** The University of Alabama at Birmingham Heersink School of Medicine, Department of Dermatology, Birmingham, AL, USA; The University of Alabama at Birmingham Heersink School of Medicine, Department of Dermatology, Birmingham, AL, USA; The University of Alabama at Birmingham Heersink School of Medicine, Department of Dermatology, Birmingham, AL, USA; The University of Alabama at Birmingham Heersink School of Medicine, Department of Dermatology, Birmingham, AL, USA; The University of Alabama at Birmingham, Department of Dermatology, Birmingham, AL, USA; The University of Alabama at Birmingham Heersink School of Medicine, Department of Dermatology, Birmingham, AL, USA; The University of Alabama at Birmingham, Department of Dermatology, Birmingham, AL, USA

## Abstract

**Background:**

Artificial intelligence (AI) and telemedicine are rapidly changing the way dermatological care is delivered. As these tools are increasingly used in tandem, understanding how patients perceive the integration of AI across different care settings is important for responsible implementation.

**Objectives:**

To assess patient perceptions of AI in dermatology across five care modalities and examine how demographic factors influence acceptance.

**Methods:**

A cross-sectional survey was conducted among 130 adults at a US academic dermatology clinic between December 2024 and April 2025. Participants rated trust, comfort, perceived quality, privacy and confidence in equitable performance across three AI-involved modalities: standalone AI apps, AI-assisted in-person visits and AI-assisted telemedicine visits. Differences in perception outcomes across the three care modalities were analysed using repeated measures Anova. Logistic and linear regressions analysed predictors of acceptance, including age, race, skin tone, socioeconomic status, rurality and technology experience.

**Results:**

Patients strongly preferred dermatologist-involved care over standalone AI, with 73.8% trusting dermatologist-guided AI and only 1.5% trusting AI apps alone. Comfort and perceptions of equal performance across skin tones were significantly higher for telemedicine and AI-assisted visits compared with AI apps (*P* < 0.001). Darker skin tone and Black race predicted lower acceptance of AI-assisted care (*P* = 0.01 and *P* = 0.003, respectively), while greater technology familiarity predicted higher acceptance (*P* = 0.05). Comfort varied by clinical scenario, with in-person visits showing dramatically higher odds of patient comfort compared with AI apps alone [odds ratio (OR) 232.8 for new concerns, OR 137.3 for serious concerns, OR 18.4 for sensitive concerns]. AI-assisted in-person visits also showed significantly higher odds of comfort over AI apps (OR 18.4 for serious concerns, OR 3.6 for ongoing concerns).

**Conclusions:**

Patients strongly prefer AI as clinical support systems rather than autonomous decision-makers, especially for high-stakes and sensitive concerns. Differences in acceptance by race and skin tone point to the need for better representation in datasets and clearer communication about how these tools perform. Moving forward, development and implementation should emphasize clinician and patient involvement, fairness and patient choice to ensure AI is integrated into dermatology in a way that earns patient trust.

## Teaser

Artificial intelligence (AI) is entering dermatology in many forms, from standalone apps to AI-assisted in-person and telemedicine visits. This study surveyed 130 adults in an academic dermatology clinic to explore how patients perceive trust, comfort, quality and fairness across these formats. Interestingly, patients rated AI-assisted visits highest in perceived quality, yet they were most comfortable and trusting with dermatologist-led care. Patients strongly preferred dermatologist-involved care over AI alone, especially for new, serious or sensitive skin concerns. The odds of preferring in-person care over AI apps were more than 200 times higher for new conditions. While greater technology experience predicted higher acceptance, Black patients and those with darker skin tones reported significantly lower acceptance of AI-assisted care, raising concerns about equity and representation. Across all scenarios, human involvement remained key to patient comfort and trust. This study provides basis and guidance for how to develop and integrate AI into dermatologic care in ways that centre on patient preferences, transparency and fairness.

What is already known about this topic?

- Artificial intelligence (AI) and telemedicine are expanding in dermatology, offering triage, diagnosis and support across home and clinical settings.
- Patients with darker skin tones face higher diagnostic inaccuracies due to underrepresentation in AI datasets, leading to mistrust.
- Prior studies mainly assess general perceptions of AI or telemedicine, rarely comparing specific combinations or context-based preferences.

What does this study add?

- Patients strongly prefer AI as an augmenting tool, not a replacement, especially for serious, new or sensitive conditions.
- Black patients and those with darker skin tones show significantly lower AI acceptance, independent of income, education, socioeconomic status or rurality, highlighting representation concerns.
- Socioeconomic status, income, education and rurality did not predict AI acceptance.
- Patients rated AI-assisted visits highest in perceived quality but valued human connection most for comfort and trust.

The rise of artificial intelligence (AI) and telemedicine is reshaping the landscape of healthcare delivery. AI, a sector of computer science involving the automation of intelligent behaviour, can be applied in medicine through subfields like machine learning, which uses large datasets to uncover diagnostic patterns and predict clinical outcomes.^1^ In ­dermatology, AI has multiple potential applications ranging from image classification tools to decision-support ­systems that can operate in various settings from patients’ homes to clinical environments.^2^ Similarly, telemedicine has experienced rapid growth, particularly after the COVID-19 pandemic.^3^ Teledermatology, the subset of telemedicine specific to dermatology, allows for remote consultative recommendations and care prioritization through remote triage.^4^

Rather than existing as separate innovations, AI and ­telemedicine can be integrated in multiple combinations across the care continuum. These configurations range from fully autonomous AI applications used by patients at home to AI-assisted telemedicine visits to AI augmentation during in-person clinical examinations.^5^ Each modality combination may create a distinct patient experience that evokes varying levels of trust, comfort, quality perceptions and confidence in performance. Although these innovations promise potentially improved access and efficiency, implementation of these technologies should involve caution as several patient demographic factors may influence patient acceptance.^6,7^ In fact, prior research indicates that patients with darker skin tones are more likely to experience diagnostic inaccuracies due to their under-representation in AI training datasets.^8^ This lack of representation can lead to mistrust in AI technologies, potentially widening existing healthcare disparities.^8^

Understanding how patients perceive medical AI has become increasingly relevant as these technologies move towards clinical implementation. Recent work has begun to illuminate the psychological mechanisms underlying patient resistance to AI-based healthcare. Cadario *et al*. found that patients overestimate their understanding of how human physicians make medical decisions while accurately recognizing the opacity of AI processes, leading to systematic preference for human providers even when AI performance is equivalent.^9^ Longoni *et al*. identified ‘uniqueness neglect’ as an important driver of medical AI resistance, where patients worry that AI cannot adequately account for their individual characteristics and circumstances; however, in this study, this resistance was eliminated when AI was positioned as supporting rather than replacing human decision-making.^10^ In separate research, Reis *et al*. demonstrated that identical medical advice receives lower trust and empathy ratings when patients believe it originates from AI sources rather than human physicians. This negative bias occurred even when the advice was described as being generated by AI working in collaboration with human physicians, suggesting that any perceived AI involvement may reduce patient confidence.^11^

These studies provide important foundational evidence of patient resistance to medical AI. However, this topic has significant gaps in understanding how patients respond to specific care delivery configurations in real-world specialty care settings, particularly regarding how demographic characteristics and clinical condition types might influence acceptance of different combinations of AI, telemedicine and physician involvement in actual practice. Our study addresses this gap by comparing multifaceted patient perceptions across five dermatology consultation types: in-person visits, telemedicine visits, AI-assisted in-person visits, AI-assisted telemedicine visits and independent AI app consultations. We further examined how these perceptions are influenced by both patient characteristics (e.g. age, skin tone, race, income level, education level, neighbourhood-level socioeconomic status, rurality and technology experience) and the clinical context in which these technologies will be used. By exploring nuanced perceptions across modality combinations, we aimed to identify which aspects of AI involvement align with positive patient perceptions, and which elements require better transparency, representative design and thoughtful implementation to address negative patient perceptions. This granular understanding is essential for determining exactly how to alleviate patient concerns, improve trust and optimize outcomes in ways that ensure equitable access and quality as these technologies become increasingly embedded in dermatological care delivery.

## Materials and methods

This cross-sectional study surveyed adults at the University of Alabama at Birmingham outpatient dermatology clinic between 20 December 2024 and 16 April 2025. Participants were recruited using consecutive sampling during afternoon general dermatology clinic sessions, with all adult patients present during recruitment periods approached for ­participation. The sample included both new and follow-up dermatology patients, with the majority being follow-up patients with established dermatological care relationships. Participants provided written informed consent and completed surveys either electronically via Qualtrics (https://www.qualtrics.com/) or on paper.

### Survey development

Survey items were adapted from three validated instruments: the Technology Acceptance Model,^12^ the Patient Trust in Medical Technology Scale^13^ and the Patient Perceptions of AI and Telemedicine in Dermatology framework, with questions adapted from Wu *et al*. and Maul *et al*.^14,15^ The survey underwent expert review by an interdisciplinary panel of eight members, including four dermatologists, two epidemiologists and two professors specializing in AI in medicine. The survey was calibrated to a grade 4–5 reading level. Pilot testing (*n* = 5) refined question interpretation and flow. The complete survey instrument is provided in Supplementary File S1 (see Supporting Information).

### Outcome measures

Participants rated anticipated trust, comfort, perceived care quality, privacy and confidence in equitable performance across skin tones for three care types: telemedicine, AI-assisted dermatology visits and standalone AI apps. Clear definitions were provided to participants for each care type: telemedicine was defined as ‘talking to your dermatologist using video calls or messages instead of going to the office’; AI-assisted visits were defined as ‘during visits with your dermatologist (in-person or through video calls), AI helps the dermatologist by taking notes, looking at pictures of your skin, and/or giving ideas to help with your care’; and AI apps were defined as ‘apps that use AI to look at pictures of your skin and give you advice or ­information’ with explicit clarification that ‘these apps do NOT involve a dermatologist’. Responses used a 4-point Likert scale (1 = strongly disagree; 4 = strongly agree) to discourage neutral responses. Additionally, participants evaluated trust preferences across hypothetical scenarios where they received conflicting advice from different provider configurations. Three specific scenarios were presented: (i) comparing advice from telemedicine visits with and without AI assistance, (ii) comparing advice from in-person visits with and without AI assistance, and (iii) comparing advice from a dermatologist using AI assistance vs. a standalone AI app. For each scenario, participants indicated which source they would trust more, with options including preferring one source, trusting both equally or trusting neither option. Additional items assessed general technology experience (0–10 scale), experience and/or familiarity with the three dermatology care types assessed (telemedicine, AI-assisted dermatology visits and standalone AI apps), demographics and zip-code-linked Distressed Community Index (DCI) scores ranging from 1 (most prosperous) to 100 (most distressed).^16^ Skin tone was grouped according to Fitzpatrick skin phototype: I/II (group 1), III/IV (group 2) and V/VI (group 3). Technology experience was rated subjectively by patients out of 10, with 10 being the most experienced. Rurality was assessed using Rural-Urban Commuting Area (RUCA) codes,^17^ with scores >3 classified as rural and ≤3 as urban per the Health Resources and Services Administration.^18^

### Statistical methods

Before data collection, a power calculation was performed. Assuming a moderate effect size, α = 0.05 and 80% power, the required sample size was estimated to be approximately 127 participants for the statistical analyses planned. A moderate effect size was chosen as appropriate given both statistical considerations and the practical feasibility of collecting patient responses within the study time frame. Our final sample of 130 therefore met this target. Descriptive statistics were calculated for all demographic and survey response variables. Trust preferences across hypothetical scenarios were described using frequencies and proportions. Differences in perception outcomes across the three care modalities were analysed using repeated measures Anova. Post hoc pairwise comparisons were conducted using Bonferroni correction to account for multiple comparisons. To assess predictors of participant acceptance of modalities, multivariable linear regression models were performed for each care modality. Independent variables included age, race, skin tone, DCI score, rurality, education levels, income levels and technology experience. Multicollinearity was assessed using variance inflation factors (<5) and homoscedasticity through residual plots; all assumptions were met. Binary logistic regression examined comfort preferences across five clinical scenarios (minor, serious, new, ongoing and sensitive area concerns) using AI apps as the reference. All analyses used SPSS version 30.0 (IBM, Armonk, NY, USA) with significance at *P* < 0.05.

## Results

The response rate was 69.1% (*n* = 130/188). Of the 130 completed surveys, 113 (86.9%) were completed electronically via Qualtrics and 17 (13.1%) were completed on paper. The mean (SD) age of the participants was 45.8 (18.3) years; 55.4% (*n* = 72) were women. The majority of participants identified as White (*n* = 75; 57.7%) or Black (*n* = 45; 34.6%). Skin tones were fairly distributed across the spectrum, from Fitzpatrick skin phototype I (*n* = 10; 7.7%) to VI (*n* = 9; 6.9%). The majority (*n* = 75; 57.7%) of participants had a bachelor’s degree or higher. Participants were also economically diverse, with household incomes spanning from <$50 000 (*n* = 31/127; 24.4%) to ≥$100 000 (*n* = 46/127; 36.2%). The sample represented moderately distressed communities [mean (SD) DCI 54.0 (33.3)] with the majority (*n* = 111/124; 89.5%) living in urban communities and reporting high technology familiarity [mean (SD) technology experience score 8.1 (2.0)]. Regarding prior technology experience in dermatology care, 39.2% (*n* = 51/130) of participants reported familiarity with the use of telemedicine for their dermatological healthcare, 16.2% (*n* = 21/130) were familiar with AI-assisted dermatology visits (either telemedicine or in-person) and 3.8% (*n* = 5/130) were familiar with or had used AI apps for their dermatological healthcare. Over half of participants (*n* = 70/130; 53.8%) reported no familiarity with or use of any of these technologies for their skin healthcare. See Table 1 for full descriptive statistics.

**Table 1:** Participant characteristics (*n* = 130)

Characteristic	*n* (%)
Sex	
Female	72 (55.4)
Male	58 (44.6)
Age (years), mean (SD)	45.8 (18.3)
Race/ethnicity	
White	75 (57.7)
Black	45 (34.6)
Asian	3 (2.3)
Native American	1 (0.8)
Hispanic	3 (2.3)
Prefer not to say	3 (2.3)
Fitzpatrick skin phototype	
I	10 (7.7)
II	30 (23.1)
III	34 (26.2)
IV	10 (7.7)
V	37 (28.5)
VI	9 (6.9)
Education level	
High school or less	23 (17.7)
Some college	32 (24.6)
Bachelor’s degree or higher	75 (57.7)
Household income (*n* = 127)	
<$50 000	31 (24.4)
$50 000–$99 999	31 (24.4)
≥$100 000	46 (37.1)
Prefer not to say	19 (15.0)
Rural vs. urban residence (*n* = 124)	
Rural	13 (10.5)
Urban	111 (89.5)
Distressed Community Index, mean (SD)	54.0 (33.3)
Technology experience score, mean (SD)	8.1 (2.0)
Prior technology familiarity for dermatology care	
Telemedicine	51 (39.2)
AI-assisted dermatology visits (in-person and via telemedicine)	21 (16.2)
Patient-facing AI apps	5 (3.8)
None	70 (53.8)

Data are presented as n (%) unless otherwise specified. AI, artificial intelligence.

### Trust preferences across scenarios

Participants compared hypothetical scenarios where they received conflicting skin advice from two different sources and indicated which they would trust more. In both telemedicine and in-person settings, participants were nearly split between trusting dermatologist advice with AI assistance [33.1% (*n* = 43/130) and 40.0% (*n* = 52/130), respectively] vs. dermatologist advice alone [32.3% (*n* = 42/130) for both]. However, when choosing between AI-supported dermatologist advice and a standalone AI app, preferences were decisive: 73.8% (*n* = 96/130) trusted the dermatologist, while only 1.5% (*n* = 2/130) preferred the app alone.

### Repeated measures Anova results

**Figure 1:**
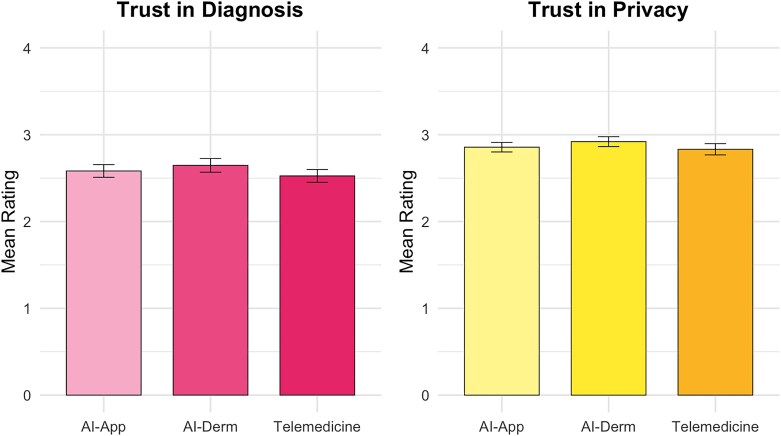
Comparison of trust in diagnosis and trust in privacy across three dermatology consultation modalities.

**Figure 2:**
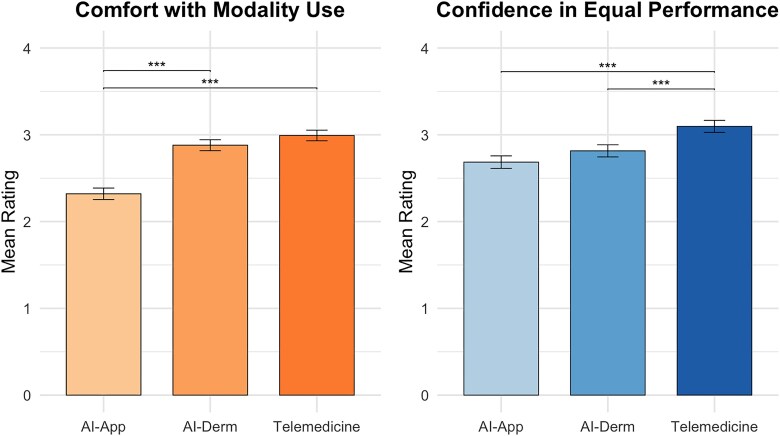
Comfort and confidence in equal performance across skin types for three dermatology consultation modalities. Post hoc analyses showed that patients were significantly more comfortable with telemedicine than with patient use of artificial intelligence (AI) apps, significantly more comfortable with AI-assisted dermatology visits than with AI apps, significantly more confident in equitable performance across skin types for telemedicine compared with AI apps and significantly more confident in equitable performance for telemedicine compared with AI-assisted visits. ***P < 0.001.

Significant differences emerged across modalities for patient perceptions of comfort (*P* < 0.001), confidence in equal performance (*P* < 0.001) and perceived quality (*P* < 0.001) but not for trust in diagnosis (*P* = 0.10) or privacy (*P* = 0.37) (Figure 1). Comfort was highest for telemedicine [mean (SD) 2.99 (0.68)], followed by AI-assisted visits [mean (SD) 2.88 (0.70)], and lowest for AI apps [mean (SD) 2.32 (0.74)]. Post hoc analyses showed that participants were significantly more comfortable with telemedicine and AI-assisted visits compared with AI apps (both *P* < 0.001) (Figure 2). Confidence in equal performance across skin types was highest for telemedicine [mean (SD) 3.10 (0.77)], followed by AI-assisted visits [mean (SD) 2.81 (0.78)] and then AI apps [mean (SD) 2.69 (0.80)], with all pairwise comparisons significant (*P* < 0.001) (Figure 2).

**Figure 3:**
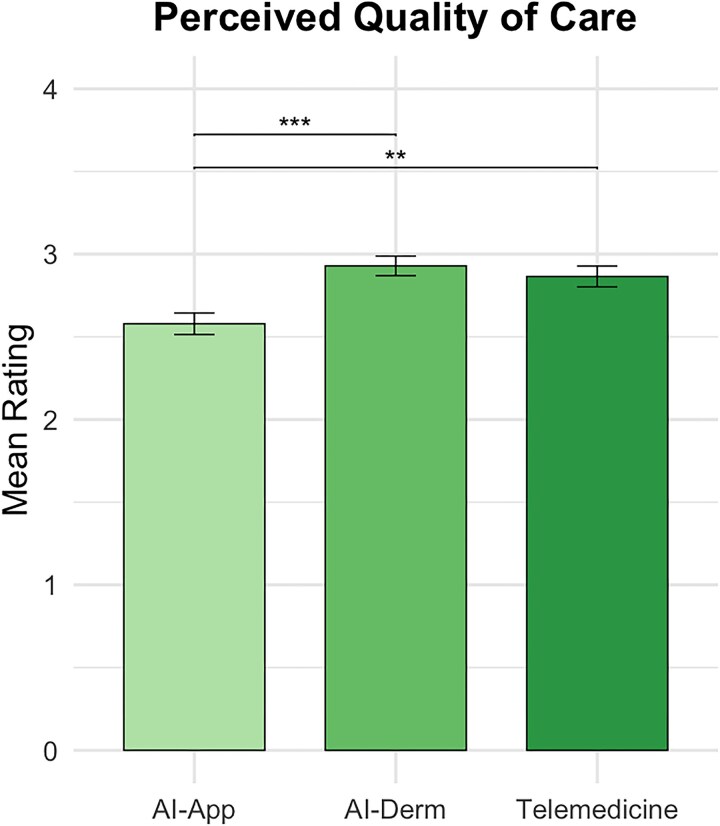
Perceived quality of care across three dermatology consultation modalities. **P < 0.05 (post hoc comparisons between modalities). ***P < 0.001 (see Figure 2).

Perceived quality was highest for AI-assisted visits [mean (SD) 2.93 (0.66)], followed by telemedicine [mean (SD) 2.87 (0.71)] and then AI apps [mean (SD) 2.58 (0.73)]. AI apps scored significantly lower than both other modalities (*P* ≤ 0.002) (Figure 3). In Figures 1–3, ‘AI-App’ refers to a standalone AI app used independently by the patient at home and ‘AI-Derm’ refers to AI assisting the dermatologist during either a telemedicine or in-person visit.

### Regression analyses results

Multivariable linear regression models were conducted separately for each consultation modality to assess predictors of participants’ overall acceptance of each modality. Each model included age, income group, education level, rurality (RUCA), DCI, race, skin tone and technology experience (Table 2). The model for AI-assisted dermatology visits was statistically significant [*F*(9115) = 2.14, *P* = 0.03]. Identifying as Black (β = −0.514, *P* = 0.003) and having a darker skin tone (β = −0.245, *P* = 0.01) were significantly associated with lower overall acceptance scores. In contrast, greater technology experience (β = 0.029, *P* = 0.05) was associated with more favourable perceptions. Notably, age, income, education level, rurality and community economic distress (DCI) were not associated with acceptance of AI use by dermatologist (Table 2). The models for telemedicine [*F*(9114) = 1.68, *P* = 0.10] and AI apps without dermatologist involvement [*F*(9115) = 0.94, *P* = 0.49] were not statistically significant, and no consistent predictors emerged across these modalities.

**Table 2:** Significant predictors from multivariable regression models of positive patient perceptions of artificial intelligence helping dermatologists

Predictor	β	95% CI	*P*-value
Age	0.003	–0.007, 0.012	0.29
Income group	0.020	–0.033, 0.074	0.44
Education group	–0.029	–0.174, 0.115	0.70
DCI	0.000	–0.006, 0.007	0.79
Rurality (RUCA)	0.023	–0.307, 0.353	0.89
Technology experience	0.029	0.001, 0.057	**0**.**05**
Skin tone	–0.245	0.059, 0.431	**0**.**01**
Race (Black)^a^	–0.514	–0.851, –0.178	**0**.**003**
Race (Hispanic)^a^	–0.224	–0.893, 0.445	0.51
Race (Asian)^a^	–0.458	–1.158, 0.242	0.20
Race (American Indian or Alaska Native)^a^	–0.759	–1.772, 0.253	0.98

CI, confidence interval; DCI, Distressed Community Index; OR, odds ratio; RUCA, Rural-Urban Commuting Area code. ^a^Reference group is White. For the overall linear regression model: [*F*(11,115) = 2.14, *P* = 0.03]. Bold values denote statistical significance (*P* < 0.05).

### Logistic regression results

**Figure 4:**
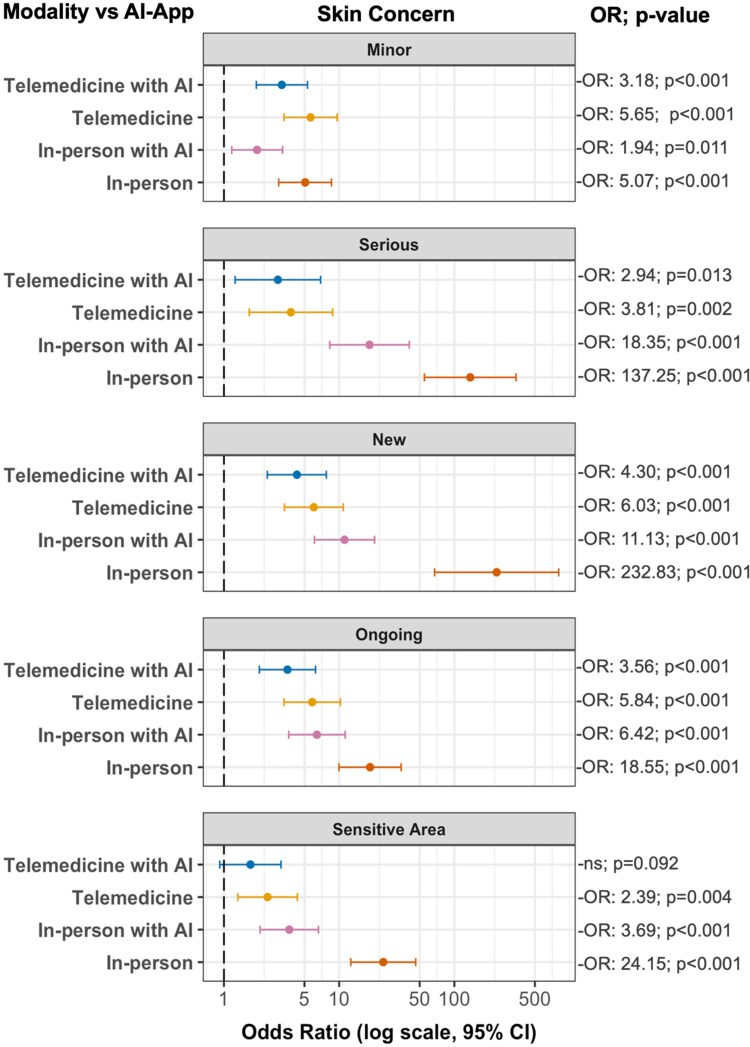
Odds ratios for patient comfort with different dermatology consultation modalities.

Binary logistic regressions examined the association between consultation modality and patient-reported comfort across different skin condition types, using AI app consultation as the reference group (Figure 4). The findings revealed that consultation modality preferences varied dramatically by condition type, with all associations significant (*P* < 0.001). For minor problems, participants showed modest preferences for all modalities over AI apps [odds ratio (OR) 1.94–5.65], with the strongest preference for standard telemedicine (OR 5.65). However, as condition severity increased, preferences for human involvement intensified dramatically. For serious conditions, traditional in-person visits showed 137-fold higher odds of comfort than AI apps (OR 137.25, *P* < 0.001), while the OR range for other modalities was 2.94–18.35. New skin concerns elicited even stronger preferences for traditional care (OR 232.83 vs. AI apps, *P* < 0.001). Ongoing problems showed intermediate preferences (OR 3.56–18.55), while sensitive area concerns were notable for being the only scenario where AI-assisted telemedicine was not significantly preferred over AI apps (OR 1.70, *P* = 0.09). Across all scenarios, traditional in-­person visits consistently ranked highest for comfort, with AI-assisted in-person visits ranking second for all but minor conditions, and preference magnitudes scaling dramatically with perceived condition importance (ORs ranging from 5.07 for minor issues to 232.83 for new concerns).

## Discussion

In this study, we examined patient perceptions across five distinct dermatology consultation modalities, comparing traditional approaches with various combinations of AI and telemedicine integration. Our findings reveal that patient acceptance of these technologies is multifaceted and contextual. Three major factors influenced patient perceptions of AI involvement in their care: visible human involvement in the care process, specific clinical context, and demographic factors such as skin tone, race and technology familiarity. These nuanced findings have significant implications for future development and implementation of AI and telemedicine in dermatology, suggesting that integration strategies must consider not only technological capabilities, but also patient preferences, clinical contexts and equity concerns.

Our findings demonstrate that across consultation types, trust in care remains strongly anchored to the involvement of a human dermatologist. This finding was particularly true when AI was used to support rather than replace clinical judgement. During in-person visits, more participants trusted dermatologists’ advice when augmented by AI (40.0%) than advice from dermatologists alone (32.3%), suggesting that patients may view AI as a tool that supports rather than replaces clinical judgement. However, this trust dynamic shifted in the context of telemedicine visits, where confidence in dermatologist advice with AI was 33.1% compared with 32.3% for dermatologist advice without AI, indicating nearly identical levels of trust. This contrast could indicate that patients may be more cautious about AI’s value benefit when relational cues are absent from the care setting. These findings support the principle of augmented intelligence, where AI complements but does not supplant the clinician’s role in decision-making.^19^ Notably, when participants were asked to choose between dermatologists’ advice supported by AI and an AI app alone, 73.8% preferred the dermatologist with AI, while only 1.5% favoured the app alone. This contrast highlights patient preference for human accountability in care delivery. Prior studies support these findings. In a survey by Wu *et al*., 84.4% (*n* = 119/141) of participants trusted dermatologist diagnoses over AI alone, and 68.1% (*n* = 96/141) of patients preferred care delivered by AI and a dermatologist in combination over isolation of either.^14^

Patient preference in dermatological care also varied substantially by the clinical context. Logistic regression models revealed that patient trust and comfort were strongly associated with perceived risk of the condition. As the seriousness or sensitivity of a condition increased, so did the demand for human involvement. For serious, new or sensitive concerns, the odds of preferring traditional in-person visits over AI apps increased dramatically, up to 233 times higher for new conditions and 137 times higher for serious conditions. Even for sensitive and ongoing concerns, the OR for preferring traditional in-person visits over AI apps alone was far above the odds of preference for other modalities. While preferences were most pronounced in higher stake cases, patients still favoured human-led care for minor and ongoing concerns, although to a lesser degree. Interestingly, for minor conditions, patients were more likely to prefer telemedicine (OR 5.65) over traditional in-person care (OR 5.07) vs. AI apps alone. This finding suggests patients have a greater openness to remote modalities when the perceived risk is low. Prior studies reinforce the context-driven trust dynamic we found in our results. Maul *et al*. found that patients were open to telemedicine for minor issues but hesitant for severe conditions,^15^ while Ford *et al*. reported preferences for in-person care in chronic disease management.^20^ Notably, patients showed strong support for AI-assisted in-person visits as the second-most preferred option across nearly all scenarios. This indicates that patients may believe that AI can enhance care even in high-risk cases, as long as dermatologists remain central to decision-making. This challenges existing literature that advocates for AI as primarily suitable for triage in low-risk scenarios. Salinas *et al*. noted that most dermatological AI tools are designed for basic classification and triage of benign conditions in low complexity settings.^21^ Instead, our findings support a more nuanced role for AI as a clinical extender across a broader spectrum of concerns. Considered together, while patients are open to more flexible care options for low-risk concerns, they may still expect direct involvement from a dermatologist when the condition feels serious or personal.

Our findings reveal persistent disparities in how AI in dermatology is perceived across racial and skin tone groups. Identifying as Black or having a darker skin tone were independently associated with lower overall perception scores regarding AI assistance in dermatological care, even after adjusting for income, education, rurality and technology experience (*P* = 0.003 and *P* = 0.01, respectively) (Table 2). Conversely, participants with greater technology experience reported more favourable perceptions (*P* = 0.05) (Table 2). This finding aligns with prior studies showing that familiarity with AI can increase patient comfort and willingness to engage (Wu *et al*. 2022; Sangers *et al*. 2021).^14,22^ Our results support this conclusion while also considering that such familiarity may not eliminate concerns among Black patients or individuals with darker skin types. These findings reflect longstanding concerns about under-representation in dermatological datasets and the potential for algorithmic bias.^8,23^ Our findings suggest that acceptance of AI in dermatology is shaped not only by exposure, but also by skin tone and race, reiterating the need for diverse, representative datasets and transparent reporting of AI performance across skin types. Our Anova results provide more granularity into participant perceptions of bias in AI. Participants expressed less confidence in AI performing equally well across skin tones when compared with telemedicine, both in settings where AI helps the dermatologist and when AI was used independently through apps (Figure 2). The lack of trust that persisted even when a dermatologist was involved may imply that human presence alone does not resolve concerns about bias. Together, these findings reinforce ­equity-based concerns that contribute to hesitancy toward AI implementation in dermatology.

Interestingly, remote or in-person visits where AI helps the dermatologist were rated highest in perceived quality of care [mean (SD) 2.93 (0.66)], surpassing both AI apps used by patients at home and standard telemedicine encounters (Figure 3). This suggests that patients recognize AI’s potential benefit in clinical dermatology when properly integrated. These findings are supported by prior work. In a survey performed by Goessinger *et al*., 95.5% (*n* = 192/201) of patients believed AI improved diagnostic accuracy,^24^ while Nelson *et al*. found that patients associated AI use with faster triage and improved workflow.^2^ However, this perceived improvement in quality did not translate to significantly greater trust in diagnostic accuracy (*P* = 0.10) or data privacy (*P* = 0.37) in AI-augmented visits (Figure 1). Additionally, participants expressed the highest comfort levels with standard telemedicine, not AI-augmented care (Figure 2). This discrepancy suggests that while AI may be perceived as clinically beneficial, patients continue to prioritize familiarity, relational dynamics and personal connection when it comes to comfort and trust.

No significant associations were found between patient perceptions of AI assistance at dermatology visits and common socioeconomic indicators, such as age, income, education level, DCI score or rural status (Table 2). These variables are frequently cited in discussions of digital health disparities, where structural barriers such as broadband access and digital literacy are often presumed to influence perception and engagement.^25,26^ Our findings offer a novel contribution to the emerging literature by suggesting that these commonly referenced structural determinants may not be primary predictors of AI acceptance in this domain. For many patients, scepticism toward AI may be less about structural access and more about relational or experimental elements as we have discussed throughout. Recognizing that distinction will be valuable for patient-centred implementation frameworks.

It is important to emphasize that while several AI tools for dermatology have received regulatory approval, most remain limited in their widespread clinical implementation. At the time of writing, only three AI-powered dermatological devices have secured Food and Drug Administration approval: MelaFind (discontinued in 2017), Nevisense and DermaSensor. DermaSensor received Food and Drug Administration Class II approval in January 2024 for use by primary care physicians.^27^ Internationally, several AI apps have received Conformité Européenne (CE) marking in Europe, including SkinVision, Deep Ensemble for the Recognition of Malignancy, and Skinive, although their clinical adoption varies significantly.^27^ Despite these approvals, widespread integration into routine dermatological practice remains limited. As these technologies move toward broader clinical adoption, our findings offer a roadmap for implementation strategies that align with real patient preferences and equity considerations. This study provides multifaceted insights about various ways AI could be integrated into patient care – from AI-assisted telemedicine and in-­person visits to standalone AI applications – all of which evoke distinctly different patient experiences and acceptance levels. Specifically, the data support a model of graduated, patient-centred AI integration where patients expect AI to enhance but not replace human care.

Overall, successful AI implementation in dermatology requires that practices emphasize these tools as clinical support systems, not as autonomous decision-makers. Additionally, AI models that use representative, diverse datasets and disclose any inequity across skin types should be prioritized.^8^ Visible human oversight and clear clinician accountability may increase patient trust and acceptance. Importantly, patients should be offered choices in how AI is used during their care, with decisions guided by their comfort rather than convenience or efficiency alone. Beyond informing clinical implementation strategies, our findings highlight the importance of incorporating patient perspectives during AI development itself. Our detailed data on patient preferences across clinical contexts, demographic groups and care configurations could guide developers to create patient-centred tools from the outset. Specifically, developers could collaborate with diverse patient populations as stakeholders during the design phase to ensure AI tools align with patient care preferences and address equity concerns. This approach may foster AI development that achieves both technical excellence and meaningful patient trust to ultimately advance equitable healthcare innovation.

This study has several limitations. The cross-sectional design limits our ability to assess how patient perceptions of AI may evolve over time. Additionally, while using a 4-point Likert scale to discourage neutral responses can reduce response bias and improve our ability to detect meaningful differences,^28,29^ this approach may have introduced other forms of bias by compelling participants to express preferences they may not genuinely hold, particularly when participants truly lacked strong opinions about AI technologies. The use of hypothetical scenarios, while allowing systematic comparison across modalities, may not fully capture how patients would respond to actual clinical encounters with these technologies. Additionally, while our survey questions asked participants to evaluate care configurations where AI supported rather than replaced dermatologists, some questions (particularly trust scenarios comparing AI-assisted dermatologists vs. standalone AI apps) could be susceptible to social desirability bias or demand effects when participants might feel pressure to favour options with physician involvement. Furthermore, the subjective nature of ‘quality of care’ perceptions may have introduced variability in interpretation, as participants likely drew from diverse personal experiences and values when evaluating this construct. Our sample demonstrated age-related selection bias, with 62% (*n* = 36/58) of nonparticipants being older than 50 years of age. However, age was not a significant predictor of positive patient perceptions of AI-assisted dermatology visits and was controlled for in our linear regression (Table 2). Additionally, our sample was recruited exclusively from an academic medical centre, which may introduce responder bias and limit generalizability in community-based dermatology practices where patient populations and technology adoption patterns may differ. Finally, while we noted that our sample included both new and follow-up dermatology patients with the majority being follow-up patients, we did not systematically collect the exact proportion of each patient type or analyse how patient status (new vs. established) might influence AI acceptance. Future research should build on our findings. A longitudinal design could track shifts in perception as AI becomes more integrated in dermatology. Also, qualitative interviews would provide deeper insights into reasoning behind demographic differences in acceptance. As AI becomes more integrated, implementation studies could examine whether shared modality decisions improve satisfaction, adherence or outcomes. Future studies should directly assess how patients understand complex concepts like ‘quality,’ ‘bias’ and ‘equity’ in the context of AI-driven dermatological care. Future research could additionally examine whether patients with different levels of experience with dermatological care exhibit varying comfort levels with AI integration. Additionally, although our overall power target was met, detecting smaller effect sizes would require larger sample sizes, which future studies should pursue to refine these findings. Finally, while repeated measures Anova was appropriate for evaluating group-level differences across modalities in this study, future work with larger samples and expanded statistical expertise could employ mixed-effects modelling to more fully account for random effects such as scenario-level variation.

This study offers a detailed assessment of patient perceptions of AI integration across dermatology care modalities, showing that patient acceptance of AI in dermatology is shaped less by technology itself and more by how it is integrated into care. Patients showed the greatest trust and comfort when AI supported, rather than replaced, dermatologists – especially in high-stakes or sensitive situations – underscoring the enduring importance of human connection in healthcare. While prior exposure to technology improved perceptions, Black patients and those with darker skin tones reported lower acceptance, reflecting deeper concerns about fairness and representation surrounding AI. As AI tools become more common in dermatology, development and implementation must centre not only on innovation, but on transparency, inclusivity and patient choice to ensure these advances truly serve all patients, regardless of background.

## Supplementary Material

vzaf086_Supplementary_Data

## Data Availability

The datasets generated and analysed during the current study are not publicly available due to participant confidentiality and privacy considerations but are available from the corresponding author upon reasonable request and with appropriate ethics approval.
