# Supplementary material for: Patient perceptions of artificial intelligence integration in dermatology: a cross-sectional study of trust, comfort and equity across multiple care modalities
Source: Skin Health Dis. 2025 Dec 22;6(1):35–44. doi: 10.1093/skinhd/vzaf086 (PMC12867938; doi:10.1093/skinhd/vzaf086)
Supplement: vzaf086_Supplementary_Data [file vzaf086_supplementary_data.docx]

### **Section 1: Demographic Information**

1. **What is your age? ______**
2. **What is your gender?**

- Male
- Female
- Prefer to self-describe: _________
- Prefer not to say

1. **What is your highest level of education completed?**

- High school or less
- Some college
- Bachelor's degree
- Graduate or professional degree
- Prefer not to say

1. **What is your annual household income?**

- Less than $25,000
- $25,000 - $49,999
- $50,000 - $74,999
- $75,000 - $99,999
- $100,000 - $149,999
- $150,000 or more
- Prefer not to say

1. **What is your race/ethnicity? (Select all that apply)**

- White/Caucasian
- Black/African American
- Hispanic/Latino
- Asian/Pacific Islander
- Native American/Alaska Native
- Other (please specify): __________
- Prefer not to say

1. **What is your ZIP code? __________**

- *Providing your ZIP code will help us look at regional differences on thoughts about skin technologies. Your ZIP code will be kept confidential and used solely for research purposes.*

1. **How would you classify your skin type? (Please select one)**

- **Very Fair**: Very light skin. Burns quickly, almost never tans.
- **Fair**: Light pink or beige skin. Burns easily, tans slowly.
- **Medium**: Light pink or beige skin. Sometimes burns, tans gradually.
- **Olive**: Light brown skin. Hardly ever burns, tans easily.
- **Brown**: Medium to dark-brown skin. Rarely burns, tans quickly.
- **Dark**: Very dark-brown skin. Almost never burns, tans a lot.

### **Section 2: Familiarity and Prior Experience**

**Definitions:**

- ***Telemedicine***:
  - Talking to your dermatologist using video calls or messages instead of going to the office.
- ***Visit Where AI Helps Your Dermatologist****:*
  - During visits with your dermatologist (in person or through video calls), AI helps the dermatologist by taking notes, looking at pictures of your skin, and giving ideas to help with your care.
- ***AI Apps****:*
  - Apps that use AI to look at pictures of your skin and give you advice or information. ***These apps do NOT involve a dermatologist***.

1. **Please rate your experience level from 0-10 with using technology (ex: smartphones, computers, tablets, or other digital devices)?**

Write your rating number 0-10: _______

*0=Not at all experienced
10=Extremely Experienced*

1. **Which of the following technologies are you familiar with or have used/experienced for your skin healthcare? (Select all that apply).**

- Telemedicine
- Visits where AI helps your dermatologist (over telemedicine or in-person)
- AI apps on my own for skin concerns
- None of the above

1. Please indicate how much you agree or disagree with each statement about **telemedicine** in the following table.

| **Statement** | **Strongly Disagree** | **Disagree** | **Agree** | **Strongly Agree** |
| --- | --- | --- | --- | --- |
| I trust the diagnoses and treatment plans my dermatologist gives during telemedicine visits. | ☐ | ☐ | ☐ | ☐ |
| I feel comfortable using telemedicine for my skin healthcare. | ☐ | ☐ | ☐ | ☐ |
| Telemedicine can improve the quality of skin healthcare I receive. | ☐ | ☐ | ☐ | ☐ |
| I trust that my personal health information remains private and secure when I use telemedicine. | ☐ | ☐ | ☐ | ☐ |
| I feel confident that telemedicine works accurately and equally well for all skin tones. | ☐ | ☐ | ☐ | ☐ |

1. Please indicate how much you agree or disagree with each statement about **AI helping your dermatologist**.

| **Statement** | **Strongly Disagree** | **Disagree** | **Agree** | **Strongly Agree** |
| --- | --- | --- | --- | --- |
| I would trust in the diagnoses and treatment plans if AI were used to help my dermatologist. | ☐ | ☐ | ☐ | ☐ |
| I would feel comfortable knowing that AI is working in the background during my visits. | ☐ | ☐ | ☐ | ☐ |
| AI helping my dermatologist during my visit would improve the quality of care I receive. | ☐ | ☐ | ☐ | ☐ |
| I trust that my personal health information would remain private and secure when AI is used by my dermatologist. | ☐ | ☐ | ☐ | ☐ |
| I feel confident that if my dermatologist uses AI, they would provide accurate care that works equally well for all skin tones. | ☐ | ☐ | ☐ | ☐ |

1. Please indicate how much you agree or disagree with each statement about **AI apps**.

| **Statement** | **Strongly Disagree** | **Disagree** | **Agree** | **Strongly Agree** |
| --- | --- | --- | --- | --- |
| I trust that AI apps can provide accurate advice about my skin without a dermatologist. | ☐ | ☐ | ☐ | ☐ |
| I feel comfortable using AI apps on my own to check my skin. | ☐ | ☐ | ☐ | ☐ |
| Using AI apps on my own can improve how I take care of my skin. | ☐ | ☐ | ☐ | ☐ |
| I trust that my personal health information remains private and secure when using AI apps for my skin health. | ☐ | ☐ | ☐ | ☐ |
| I feel confident that AI apps for skin health work equally well for all skin tones. | ☐ | ☐ | ☐ | ☐ |

1. For each skin problem listed below, please **select all technologies** you would feel comfortable using.

| **Skin Problem** | **Telemedicine** | **Telemedicine where AI helps the doctor** | **Traditional in-person visit** | **In-person visit where AI helps the doctor** | **AI apps that I use on my own** |
| --- | --- | --- | --- | --- | --- |
| New skin problem | ☐ | ☐ | ☐ | ☐ | ☐ |
| Ongoing skin problem | ☐ | ☐ | ☐ | ☐ | ☐ |
| Serious skin problem | ☐ | ☐ | ☐ | ☐ | ☐ |
| Minor skin problem | ☐ | ☐ | ☐ | ☐ | ☐ |
| Skin problems in sensitive areas | ☐ | ☐ | ☐ | ☐ | ☐ |

1. **Imagine you received different advice about your skin. For each scenario below, select which option you would trust more.**

**Scenario 1**: You are given different advice during a telemedicine visit ***without AI*** and a telemedicine visit ***with*** ***AI***.

- I would trust the dermatologist the most during the telemedicine visit ***without AI*** assistance.
- I would trust the dermatologist the most during the telemedicine visit ***with AI*** assistance.
- Both equally--I would trust them the same
- Neither—I wouldn't trust either option

**Scenario 2**: You are given different advice during an *in-person visit****without AI***helping your dermatologist and an in-person visit ***with*** ***AI*** helping your dermatologist.

- I would trust the dermatologist the most during the in-person visit ***without AI*** assistance
- I would trust the dermatologist the most during the in-person visit ***with AI*** assistance
- Both equally—I would trust them the same
- Neither—I wouldn't trust either option

**Scenario 3**: You are given different advice from a dermatologist who was helped by AI and an AI app you used on your own for your skin health.

- I would trust the dermatologist using AI assistance the most
- I would trust the AI app I’m using on my own the most
- Both equally—I would trust them the same
- Neither—I wouldn't trust either option.
